# The impact of the Trauma Triage App on pre-hospital trauma triage: design and protocol of the stepped-wedge, cluster-randomized TESLA trial

**DOI:** 10.1186/s41512-020-00076-1

**Published:** 2020-06-18

**Authors:** Rogier van der Sluijs, Audrey A. A. Fiddelers, Job F. Waalwijk, Johannes B. Reitsma, Miranda J. Dirx, Dennis den Hartog, Silvia M. A. A. Evers, J. Carel Goslings, W. Margreet Hoogeveen, Koen W. Lansink, Luke P. H. Leenen, Mark van Heijl, Martijn Poeze

**Affiliations:** 1 Department of Surgery, Maastricht University Medical Center, Maastricht, The Netherlands; 2 Department of Surgery, Utrecht University Medical Center, Utrecht, The Netherlands; 3 Network Acute Care Limburg, Maastricht University Medical Center, Maastricht, The Netherlands; 4 Department of Epidemiology, Julius Center, University Medical Center Utrecht, Utrecht, The Netherlands; 5 Department of Surgery, Erasmus Medical Center, Rotterdam, The Netherlands; 6 Care and Public Health Research Institute, Maastricht University, Maastricht, The Netherlands; 7 Department of Surgery, Amsterdam University Medical Center, Amsterdam, The Netherlands; 8 Department of Surgery, Onze Lieve Vrouwe Hospital, Amsterdam, The Netherlands; 9 Ambulance Care the Netherlands, Zwolle, The Netherlands; 10 Department of Surgery, Elisabeth TweeSteden Hospital, Tilburg, The Netherlands; 11 Department of Surgery, Diakonessenhuis Utrecht/Zeist/Doorn, Utrecht, The Netherlands

**Keywords:** Triage, Trauma Triage App, Prediction model, Emergency Medical Services, Ambulance, Impact, Trial, Stepped-wedge, Cluster-randomized, Unidirectional crossover

## Abstract

**Background:**

Field triage of trauma patients is crucial to get the right patient to the right hospital within a particular time frame. Minimization of undertriage, overtriage, and interhospital transfer rates could substantially reduce mortality rates, life-long disabilities, and costs. Identification of patients in need of specialized trauma care is predominantly based on the judgment of Emergency Medical Services professionals and a pre-hospital triage protocol. The Trauma Triage App is a smartphone application that includes a prediction model to aid Emergency Medical Services professionals in the identification of patients in need of specialized trauma care. The aim of this trial is to assess the impact of this new digital approach to field triage on the primary endpoint undertriage.

**Methods:**

The Trauma triage using Supervised Learning Algorithms (TESLA) trial is a stepped-wedge cluster-randomized controlled trial with eight clusters defined as Emergency Medical Services regions. These clusters are an integral part of five inclusive trauma regions. Injured patients, evaluated on-scene by an Emergency Medical Services professional, suspected of moderate to severe injuries, will be assessed for eligibility. This unidirectional crossover trial will start with a baseline period in which the default pre-hospital triage protocol is used, after which all clusters gradually implement the Trauma Triage App as an add-on to the existing triage protocol. The primary endpoint is undertriage on patient and cluster level and is defined as the transportation of a severely injured patient (Injury Severity Score ≥ 16) to a lower-level trauma center. Secondary endpoints include overtriage, hospital resource use, and a cost-utility analysis.

**Discussion:**

The TESLA trial will assess the impact of the Trauma Triage App in clinical practice. This novel approach to field triage will give new and previously undiscovered insights into several isolated components of the diagnostic strategy to get the right trauma patient to the right hospital. The stepped-wedge design allows for within and between cluster comparisons.

**Trial registration:**

Netherlands Trial Register, NTR7243. Registered 30 May 2018, https://www.trialregister.nl/trial/7038.

## Background

Pre-hospital trauma triage is crucial to match an injured patient to the optimal definitive care facility [1]. Erroneously transporting a patient requiring specialized trauma care to a lower-level trauma center could lead to a delay in definitive care and is associated with higher mortality and morbidity rates [2]. Conversely, transporting a patient not in need of specialized trauma care to a higher-level trauma center results in extra costs and overutilization of resources [3]. These key metrics to evaluate the quality of field triage in trauma systems are termed undertriage and overtriage, respectively [1]. The Dutch National Health Care Institute guidelines state that a maximum of 10% undertriage is acceptable in the Netherlands [4]. The mean undertriage across all inclusive trauma regions in the Netherlands was 31.4% in 2016 [5].

The first step of the multicomponent strategy to determine the optimal receiving facility is to identify patients in need of specialized trauma care. This is performed by Emergency Medical Services (EMS) professionals on-scene and is influenced by both the pre-hospital triage protocol and the judgment of the EMS professional. The 8th version of the National Protocol of Ambulance Services (NPAS; in Dutch *Landelijk Protocol Ambulancezorg*) is currently used by all EMSs in the Netherlands as the primary pre-hospital triage protocol. A recent study reported that strict adherence to the criteria of the NPAS would have led to an undertriage rate of 63.8%, with an overtriage rate of 7.3% in one inclusive trauma region [6]. Moreover, a systematic review did not identify any pre-hospital triage protocol that by itself complied with the target of 10% undertriage [7].

The Trauma Triage App (TTApp), a smartphone and tablet application that incorporates a prediction model, was recently developed to identify patients in need of specialized trauma care. The main function of the TTApp is to predict an individual patient’s probability of being severely injured. An advice regarding whether a patient requires specialized trauma care is then generated based on a pre-defined threshold probability. This novel approach to trauma triage was externally validated retrospectively in 6859 patients from a different EMS and was able to retain a c-statistic of 0.83 with proper calibration [8]. An undertriage rate of 11.2% with a combined overtriage rate of less than 50% can hereby be achieved, depending on the threshold probability.

Notwithstanding these promising validation results, the impact of the use of the TTApp in daily practice remains to be established. The TESLA (Trauma triagE using Supervised Learning Algorithms) stepped-wedge cluster-randomized trial was designed to evaluate whether the availability of the TTApp during field triage indeed leads to a decrease in undertriage, while preserving acceptable overtriage rates.

## Methods/design

### Study design

**Fig. 1:**
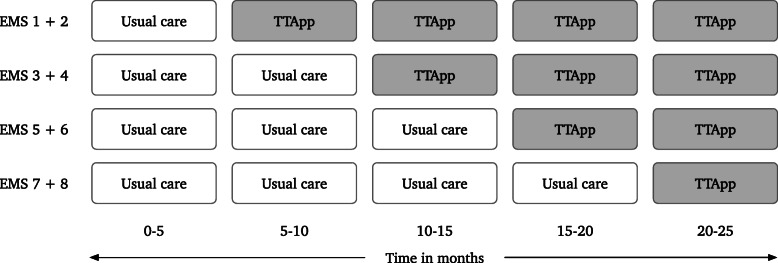
The stepped-wedge design of the TESLA-trial. Abbreviations: EMS, *Emergency Medical Service*; TTApp, *Trauma Triage App*

The TESLA trial is a prospective, stepped-wedge cluster-randomized trial (SW-CRT). In a SW-CRT, clusters are randomized into allocation sequences. These sequences all start with one or more periods under the control condition, followed by the remaining periods in which the intervention is implemented. In this trial, the participating EMS regions (the clusters) will be randomized upfront to determine the period after which two paired clusters will switch to the intervention condition. Clusters within the same inclusive trauma region were paired to ease implementation in practice. Randomization of allocation sequence was completed using computer random number generator on the primary research site (RvdS) prior to the recruitment of participants. Simple randomization was preferred to other techniques as the number of clusters was small and because there was no need to balance the influence of covariates. No methods to conceal the allocation sequence nor blinding was applied on either patient or cluster level. Our aim is to include 1920 consecutive severely injured patients in five steps, each with a duration of 4 months (details about the sample size below). All clusters will start with one or more steps of usual care (the NPAS). At the end of each step, two clusters will switch from the NPAS to the intervention condition (the TTApp used as an add-on to the NPAS). Key study design features are shown in Fig. 1.

### Participating regions

**Fig. 2:**
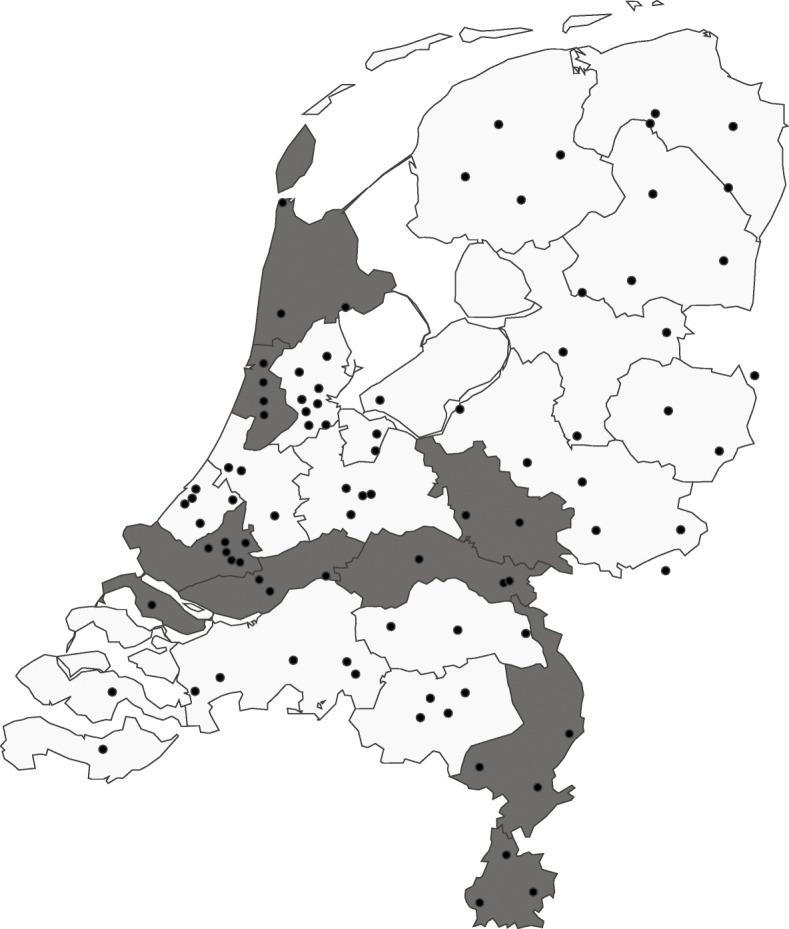
Service regions of the participating Emergency Medical Services

Eight out of 25 EMSs (the clusters) in the Netherlands, with approximately equal patient volumes, were selected to participate in this trial (Fig. 2). A combination of EMSs with heterogeneous service areas (i.e., urban, suburban, and rural) was selected to improve the generalizability of the results. All participating EMS regions take part in the Pre-hospital Trauma Triage Research Collaborative. These EMSs are an integral part of five distinct inclusive trauma regions and cover urban, suburban, and rural areas. All 37 hospitals with a trauma-receiving emergency department within these five distinct inclusive trauma regions participate in the collection of relevant patient outcomes. All hospitals with an emergency department within these regions are designated a level of care being either one, two, or three. Level one is considered a higher-level trauma center, whereas both level two and three trauma centers are acknowledged as lower-level trauma centers.

### Study population

All patients, 18 years of age or older, evaluated on-scene by an EMS professional, suspected of moderate to severe injuries, defined as an Injury Severity Score (ISS) ≥ 9, will be assessed for eligibility. Patients transported to a hospital outside of the participating trauma regions will be excluded. Patients that are dead on arrival at the initial receiving emergency department will also be excluded.

### Pre-hospital trauma triage tools

#### Usual care: National Protocol of Ambulance Services

No adjustments to daily practice of EMS professionals will be introduced prior to the switch to the intervention period. Assessment of injury severity is often a two-step process consisting of the evaluation of the pre-hospital trauma triage protocol and the final judgment of the EMS professional. The 8th version of the NPAS is currently used by all participating EMSs. This protocol is a flowchart that consists of multiple criteria to identify patients in need of specialized trauma care. If a patient fulfills one of the criteria in Table 1, the EMS professional is advised to transport the patient to a higher-level trauma center. In daily practice, it is not obligatory to adhere to the protocol or to report its advice.

**Table 1:** Higher-level trauma center criteria of the National Protocol of Ambulance Services

ABC-unstable during evaluation on-scene	
Revised trauma score < 11	
Deteriorating Glasgow Coma Scale	
Glasgow Coma Scale < 9	
Flail chest	
Amputation proximal to wrist or ankle	
Two or more fractures of the femur and/or humerus	
Penetrating injury of head, thorax, or abdomen	
Unstable pelvic fracture	
Body temperature < 32 °C	
Neurologic deficit of one or more extremities	
Anisocoria	

#### Intervention: Trauma Triage App

**Fig. 3:**
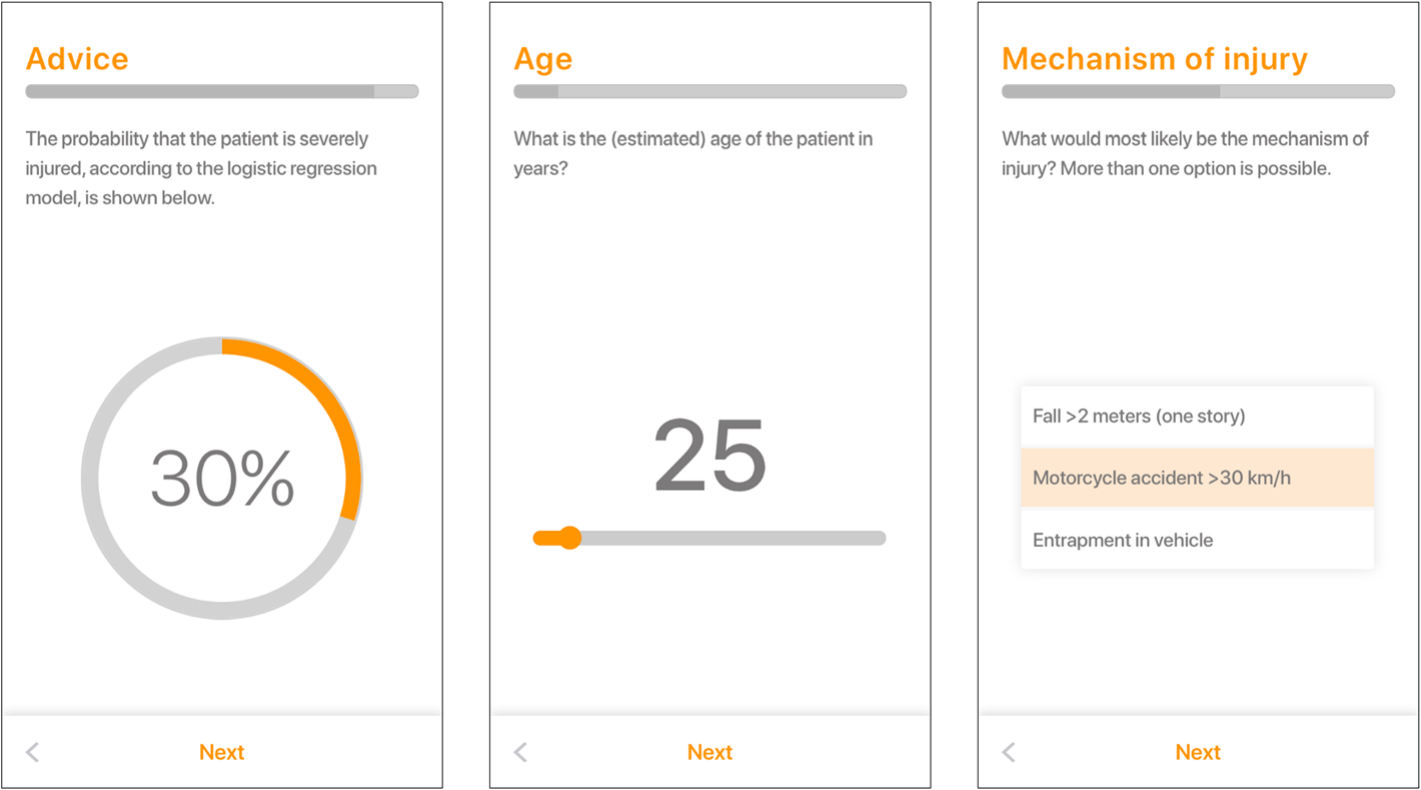
The Trauma Triage App. A sample of screens from the Trauma Triage App. Left: the generated score indicating the probability that a patient might be severely injured based on all predictors. Middle: an input field requesting the age of the patient in years. Right: an input field requesting the mechanism of injury

The TTApp is a smartphone and tablet application for both Android and iOS operating systems (Fig. 3). The application is a practical and quick-to-use questionnaire consisting of six questions that collect the required predictor values of the prediction model incorporated in the TTApp (Table 2). Four additional questions are added: the judgment of the EMS professional prior to the questionnaire, the judgment of the EMS professional after the advice returned by the prediction model, the transportation destination while displaying a map with distances to nearby hospitals, and when applicable, a screen to specify reasons to bypass the preferred hospital. The incorporated prediction model calculates the probability that a patient is severely injured. An advice whether to transport a patient to a higher-level trauma center or not is generated based on a pre-defined threshold probability. This threshold determines the sensitivity and specificity of the prediction model. Filling out the questionnaire takes approximately 30–45 s and should be performed on-scene by EMS professionals.

**Table 2:** Variables of the prediction model incorporated in the Trauma Triage App

Age	
Systolic blood pressure	
Glasgow Coma Scale	
Penetrating injury of head, thorax, or abdomen	
Fall > 2 m or motorcycle accident > 30 km/h or entrapment in vehicle	
Suspected moderate or severe head injury	
Suspected moderate or severe thoracic injury	
Injuries in at least two anatomical regions (head/neck, face, thorax, abdomen, extremities, and/or external injuries)	

Decision-making about whether to transport a patient to a higher- or lower-level trauma center will be carried out similarly to usual care, with the exception of the availability of the TTApp prediction and recommendations linked to that prediction. Also, alike the NPAS, the TTApp is a decision-support system that can be overruled by EMS professional judgment. Implementation of the TTApp will likely lead to the transportation of more (severely) injured patients to higher-level trauma centers, thus reducing undertriage. This might lead to a slight increase in overtriage.

The TTApp will be introduced to all EMSs in a systematic manner at the end of the baseline period. A presentation will be provided to all EMS professionals which teaches the rationale behind pre-hospital triage and the study protocol. An electronic-learning will be made available that will demonstrate the use of the TTApp. The application will subsequently be made available on the proprietary devices of the participating EMS. Additional teaching sessions will be organized for teams of ambulance professionals during the intervention phase in each region.

### Data collection

Pre-hospital data of trauma patients is routinely collected by EMS professionals through digital run-reports. Extra variables, mainly answers to the questionnaire and usage information, will be collected by the TTApp in the intervention period. Clinical data, including all relevant patient outcomes, of all patients admitted to one of the participating hospitals will be collected through the trauma registries of each inclusive trauma region. Injury Severity Scores will be routinely calculated within 30 days after the date of injury by trained trauma registrars for all admitted patients and those who die in the emergency department. All other included patients are assumed to have minor or moderate injuries (ISS < 16). This assumption was validated for all patients discharged from the emergency department in a previous study [6]. Hospital data and pre-hospital data will be anonymized first, and then linked using a combined deterministic and probabilistic linkage scheme. This anonymized linkage approach was validated to be both highly sensitive and specific in prior research [6]. The final dataset will be accessible by JFW, MvH, and MP.

### Patient safety

The TTApp is a diagnostic intervention aimed at EMS professionals. Regular care by EMS professionals should not be impacted by the TTApp. The prediction model is a decision support tool that—alike the NPAS—can be overruled by EMS professionals. This model is more sensitive and less specific compared to the NPAS, meaning that severely injured patients have a higher chance to get an advice for transportation to a higher-level trauma center. It will not be mandatory for EMS professionals to use the TTApp during the intervention period.

### Primary outcome

Primary endpoint of the study is undertriage, defined as the transportation of a severely injured patient (ISS ≥ 16) transported from the scene of injury to a lower-level trauma center. This implies that on patient level, a severely injured patient can be either correctly triaged and transported to a higher-level one trauma center or incorrectly triaged and transported to a lower-level trauma center.

### Secondary outcomes

- Overtriage, defined as the transportation of non-severely injured patients (ISS < 16) from the scene of injury to a higher-level trauma center, will be evaluated on patient level.
- A non-compliance analysis will be conducted to evaluate the efficacy of the TTApp under ideal circumstances with complete adherence by all EMS professionals in the specified study population.
- Use of health care resources. A comparative analysis will be performed to evaluate the differences in hospital length of stay, number of admissions to the Intensive Care Unit, and length of stay at the Intensive Care Unit, between control and intervention conditions.
- The diagnostic accuracy of the prediction model incorporated in the TTApp will be evaluated for all eligible patients.
- A cost-effectiveness analysis will be performed alongside this SW-CRT that is described in a separate protocol.

### Statistical analyses

#### Primary analysis

The primary endpoint—undertriage—will be analyzed at a patient level using a generalized linear mixed model (GLMM). A random intercept will be introduced in the model to account for cluster differences. Time will be modeled as a categorical variable denoting the cluster step. The GLMM will be used in conjunction with the binomial distribution and the identity link, resulting in a risk difference between the control and intervention condition. A small sample correction will be applied owing to the small number of clusters [9, 10]. Bootstrapped 95% CIs will be estimated from this model. This intention-to-treat analysis will be adjusted for age, which is expected to be non-linear and thus will be modeled using restricted cubic regression splines. In addition, we will estimate relative risks and corresponding confidence intervals using a log-binomial GLMM. Missing values will be multiply imputed using a multilevel multiple imputation strategy using chained equations which accounts for cluster differences using the R package micemd [11]. All statistical analyses will be conducted in R (version 3.6.2; R Foundation for Statistical Computing) [12].

### Secondary analyses

- Overtriage is analyzed using the same strategy as the primary analysis: a GLMM using a binomial distribution and the identity link resulting a risk difference. Bootstrapped 95% CIs will be calculated, and analyses will be adjusted for age. All statistics are calculated for patients with an ISS < 16.
- The primary analysis is aimed to assess the effectiveness of the TTApp. A non-compliance analysis, using instrumental variable estimation, will be conducted to evaluate the efficacy of the TTApp in the hypothetical situation with complete adherence [13, 14].
- Healthcare resources measured on a continuous scale (e.g., length of stay) are analyzed using a GLMM with a Gaussian distribution and the identity link. Numbers of admissions are converted to proportions and analyzed similar to the primary analysis.
- The probability generated by the logistic regression model incorporated in the TTApp will be calculated for all eligible patients based on the digital run-reports and the data generated by the TTApp. Diagnostic accuracy measures, such as sensitivity, specificity, predictive values, and likelihood ratios, will be calculated and model discrimination and calibration will be assessed.

#### Sample size

The primary goal of the TESLA study is to evaluate whether implementation of the TTApp in daily practice reduces undertriage. The sample size calculation is based on this endpoint. The intra-cluster correlation was calculated using the Fleiss-Cuzick method on data from studies by Voskens and colleagues and the Dutch National Trauma Registry and was 0.098 (95% CI, 0.028–0.150) [5, 6, 15]. The proportion of expected undertriage under usual care was 0.35, whereas a decrease of 0.1 was expected during the intervention period. A decrease of 0.1 was expected based on the pre-defined threshold probability of the TTApp. With eight clusters, a power of 80%, a significance level of 0.05, and two clusters switching to the intervention after every step, at least 48 severely injured patients will have to be included per cluster per step [16, 17]. The expected power given the intra-cluster correlation interval ranges from 0.79 to 0.82 [18]. Approximately 1300–1400 severely injured patients will be transported by the participating EMSs on a yearly basis; therefore, we expect a duration of (less than) 4 months per step. Patient dropout was not expected to occur [6]. The total study time consists of a baseline period plus four additional steps, totaling 20 months in which 1920 severely injured patients should be included.

Approximately 1536 severely injured patients were included until December 1, 2019, of which 576 patients were recruited during the intervention period.

## Discussion

Getting the right patient to the right hospital within a certain time frame is becoming increasingly important with the maturation of trauma systems and centralization of resources. Costs and mortality rates can be reduced by minimizing undertriage, overtriage, and interhospital transfer rates. The optimal hospital for an individual injured patient has to be determined on-scene by a diagnostic strategy that consists predominantly of (1) identification of injured patients in need of specialized trauma care and (2) logistical considerations, such as trauma center proximity and trauma center capacity. Pre-hospital triage tools, such as the Field Triage Decision Scheme, the NPAS, and the TTApp, attempt to assist EMS professionals in the first step of this strategy. These diagnostic tools should be thoroughly tested and preferably evaluated by both external validation and impact assessment before widespread implementation in clinical practice. External validation is crucial to evaluate the true performance of a prediction model in new (external) patients. Although the previously described prediction model is externally validated already, the application of the study results in practice might uncover implementation problems, disadvantages of a digital approach, and possible improvements and might thus yield different results than expected. The aim of the TESLA trial is therefore to assess the impact of the TTApp in practice. This will likely give insights into reasons for nonadherence, reasons to overrule the prediction model and its advice, and the isolated impact of many of the components in the diagnostic strategy that lead to the determination of the most optimal hospital.

Individual randomization of patients was not deemed appropriate during field triage, especially in time-critical settings. A stepped-wedge cluster-randomized design was chosen to mitigate logistical constraints such as labor intensity (e.g., training of EMS professionals) through phased implementation of the intervention and enabled all participating EMSs to evaluate the TTApp. Stepped-wedge cluster-randomized designs are particularly used to evaluate the impact of the implementation of prediction models in clinical practice [19]. This unidirectional crossover design combines elements of before-after studies with cluster randomization and is an efficient design that enables to derive a valid answer for the research question.

This study is limited by the fact that current population values were used to determine the sample size. These values might not reflect actual event rates during the trial. Patients require an ISS ≥ 9 to be eligible for inclusion in this trial. This is based on the assumption that EMS professionals are able to differentiate between mildly injured patients and those who are severely injured, which is likely, but might not entirely resemble actual usage. This constraint was posed to limit overtriage of a clearly non-severely injured group of patients. A second limitation is the use of an exchangeability correlation structure in the analyses. Since the design of this study, methodological literature has been moving towards more complex correlation structures (e.g., discrete time decay correlation) which might be more appropriate for stepped-wedge study designs [18]. Another potential limitation of the trial is the innovativeness of the TTApp and subsequently its dissimilarity to routinely used static decision schemes. This could potentially lead to substantial nonadherence. Due to these reasons and because of the fixed length of the steps in a SW-CRT and its inextensible nature, the sample size was calculated with a conservative estimate of the expected decrease in undertriage.

## Conclusion

The TESLA trial is a SW-CRT that aims to evaluate the impact of the TTApp on the primary endpoint undertriage, as well as overtriage and hospital resource use. The smartphone application can potentially acquire new and previously undiscovered insights into several components of the strategy that leads to the determination of the optimal hospital for a specific injured patient.

## Data Availability

Study results will be disseminated via a peer-reviewed international medical journal. Designated authors have to meet all four criteria for authorship in the International Committee of Medical Journal Editors (ICMJE) recommendations.

## Abbreviations

EMS: Emergency Medical Services
NPAS: National Protocol of Ambulance Services
TTApp: Trauma Triage App
TESLA: Trauma Triage using Supervised Learning Algorithms
SW-CRT: Stepped-wedge cluster-randomized trial
ISS: Injury Severity Score
GLMM: Generalized linear mixed model
